# GRP78 expression in tumor and perinephric adipose tissue is not an optimal risk stratification marker for clear cell renal cell carcinoma

**DOI:** 10.1371/journal.pone.0210246

**Published:** 2019-01-17

**Authors:** Kunyu Shen, David A. Vesey, Robert J. Ellis, Sharon Juliet Del Vecchio, Yeoungjee Cho, Armando Teixeira-Pinto, Michael A. McGuckin, David W. Johnson, Glenda C. Gobe

**Affiliations:** 1 Centre for Kidney Disease Research, The University of Queensland, Brisbane, Australia; 2 Translational Research Institute, Brisbane, Australia; 3 Department of Nephrology, Princess Alexandra Hospital, Brisbane, Australia; 4 Centre for Kidney Research, Kids Research, Children's Hospital at Westmead, Westmead, Australia; 5 School of Public Health, Faculty of Medicine and Health, The University of Sydney, Sydney, Australia; 6 Faculty of Medicine, Dentistry and Health Science, The University of Melbourne, Melbourne, Australia; University of South Alabama Mitchell Cancer Institute, UNITED STATES

## Abstract

**Objective:**

Clear cell renal cell carcinoma (ccRCC) is the most common subtype of kidney cancer, which is difficult to treat and lacks a reliable prognostic marker. A previous study showed that the endoplasmic reticulum stress marker, glucose-regulated-protein-78 (GRP78), is a potential prognostic marker for ccRCC. The present study aimed to: (1) examine whether GRP78 was upregulated in ccRCC compared with matched non-neoplastic renal tissue; and (2) investigate whether GRP78 expression in ccRCC tissue or perinephric adipose tissue has any association with ccRCC aggressiveness.

**Methods:**

A retrospective cross-sectional study of 267 patients who underwent nephrectomy for renal tumors between June 2013 and October 2017 was conducted at Princess Alexandra Hospital, Brisbane, Australia. Software-assisted quantification of average grey value of staining intensity (staining intensity method) and proportion of positive pixels (positive pixel method) was applied to measure expression of GRP78 in archived specimens of renal tumor tissues (n = 114), adjacent non-neoplastic renal tissues (n = 68), and perinephric adipose tissues (n = 60) in participants diagnosed with ccRCC.

**Results:**

GRP78 was not upregulated in renal tumor tissue compared with paired normal renal tissue. In tumor tissue, GRP78 expression did not show any association with ccRCC aggressiveness using either quantification method. In adipose tissue, downregulation of GRP78 demonstrated poor correlation with increased probability of metastasis, with one unit increase in average grey value of GRP78 staining weakly correlating with a 17% increase in the odds ratio of metastasis (95% confidence interval: 0.99 to 1.38, p = 0.07).

**Conclusion:**

GRP78 is not valuable as a risk stratification marker for ccRCC.

## Introduction

The kidney is the twelfth most common site for primary malignancy worldwide [1]. Approximately 90% of kidney cancers are renal cell carcinoma (RCC), of which clear cell (cc) RCC is the most common variety, constituting approximately 75% of all RCC diagnoses [2]. The next most common RCC variants are papillary and chromophobe RCCs, which have a lower rate of metastasis compared with ccRCC. Common benign renal tumours include papillary adenoma, renal oncocytoma, and angiomyolipoma [3]. Although imaging modalities, such as computerised tomography (CT), are able to differentiate between malignant and benign renal tumors, this process is not completely reliable, and approximately 5–8% of lesions remain indeterminate [4]. Identification of molecular markers which are better able to identify tumors expected to have worse patient outcomes would therefore be of great value to patients and clinicians.

Endoplasmic reticulum (ER) stress markers show promising risk stratification potential for ccRCC [5]. The ER is responsible for the quality control of protein folding. Accumulation of misfolded proteins causes ER stress, which is correlated with tumorigenesis [6]. Glucose-regulated-protein-78 (GRP78) is a chaperone of the heat shock protein 70 family and is one of the best-recognised ER stress markers [7]. Fu and colleagues first reported the upregulation of GRP78 in the renal tumor tissue from 42 Chinese ccRCC patients [5], where they showed association between GRP78 expressions with clinicopathological features. There was a significantly higher expression of GRP78 in renal tumors compared to the adjacent non-neoplastic kidney, and the level of GRP78 expression was positively correlated with advanced tumor-node-metastasis (TNM) stages and larger tumor size. However, the conclusions that could be drawn from this study were limited by lack of adjustment for confounders (e.g. body mass index [BMI], diagnosis of hypertension and smoking history), possible observer bias due to the subjective nature of semi-quantitative measurement, small sample size and limited generalizability with respect to race. Moreover, the clinical translation of the finding was restricted due to its dependence on renal biopsy, which is not consistently performed in the diagnosis of kidney cancer considering the low diagnostic accuracy and various complications [8].

The biopsy of perinephric adipose tissue poses less risk for patients than renal biopsy if we could find a marker which can predict aggressiveness of ccRCC. Expression of ER stress markers in adipose tissue has been shown to be a potent prognostic marker for endometrial cancer. In a retrospective study (N = 179), Koji *et al*. [9] investigated the correlation between clinical outcomes of endometrial cancer and expression of ER stress markers in visceral adipose tissue. They found that the proportion of positive staining for GRP78 and C/EBP homologous protein (CHOP) was positively correlated with higher tumor stage (p = 0.005) and negatively associated with disease-free survival (hazards ratio/HR = 2.88, p = 0.005). Similar to the contributing role of obesity for endometrial cancer, being overweight (BMI ≥ 25kg/m^2^) is an established risk factor for developing ccRCC, with the observed relative risk being 1.5 times higher than that of patients with a normal BMI [10]. Paradoxically, overweight patients appear to have a lower risk of dying from kidney cancer (HR = 0.49) [11]. Little is known about the expression of GRP78 in perinephric adipose tissue and its potential prognostic role for ccRCC.

To further elucidate the relationship between ER stress, obesity and kidney cancer, we applied immunohistochemistry (IHC) staining to investigate GRP78 expression in renal tumor, non-neoplastic renal tissue and perinephric adipose tissue. The study hypotheses included that, 1) GRP78 expression is upregulated in tumor tissue compared with matched normal renal tissue; and 2) the expression levels of GRP78 in both tumor tissue and perinephric adipose tissue are positively associated with ccRCC aggressiveness. Understanding the expression patterns of the ER stress marker GRP78 in renal samples and perinephric adipose tissue may prove to be translationally meaningful, by allowing risk stratification of ccRCC patients and development of effective therapies which modulate ER stress levels. Moreover, exploring the prognostic role of GRP78 expression in perinephric adipose tissue would help to better understand the roles of tumor-associated adipose tissue in the pathogenesis of ccRCC.

## Methods

### Sample and data collection

**Fig 1:**
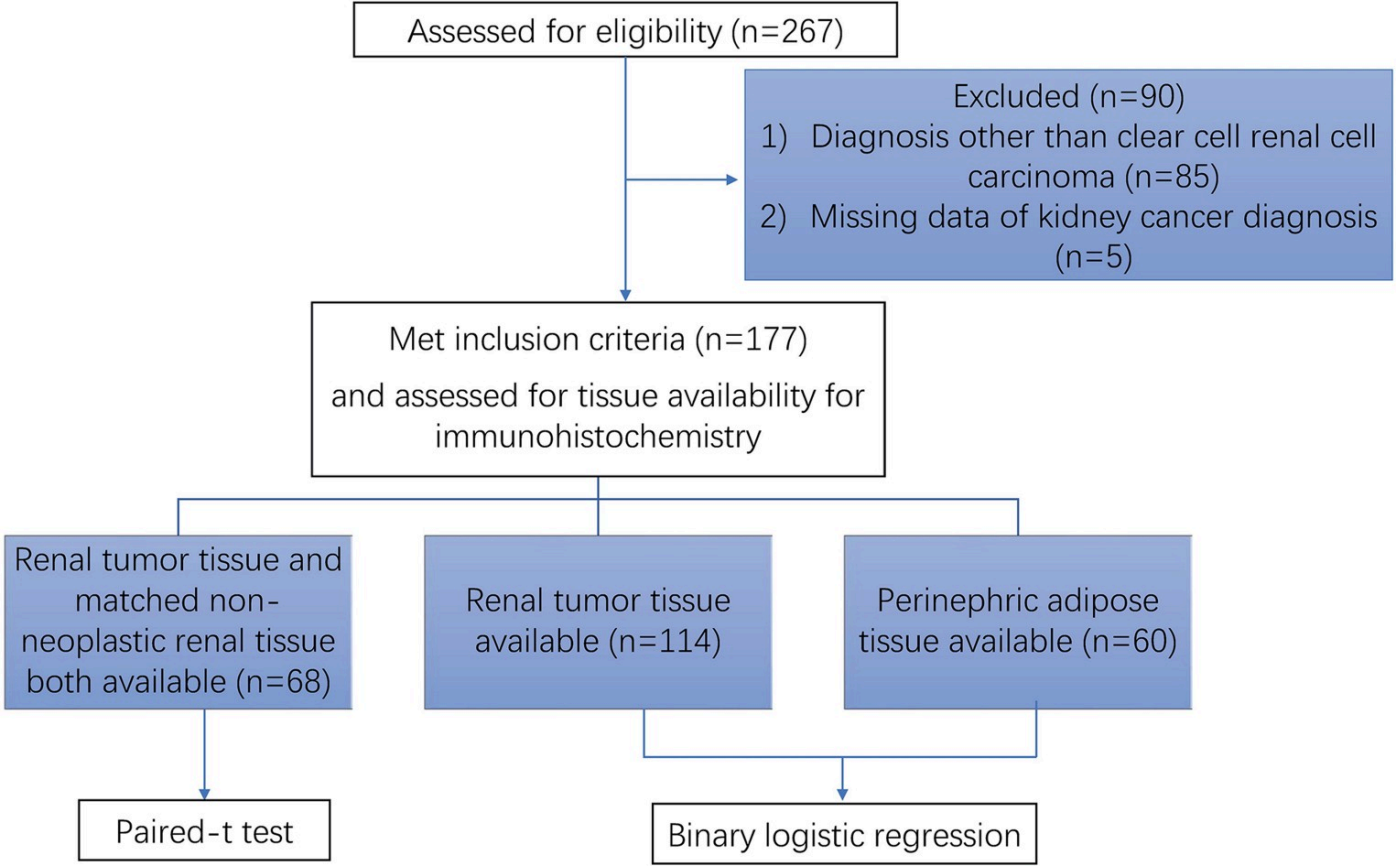
Flow chart of participation in the study. 267 participants recorded in the database were assessed for eligibility. 90 participants who did not meet the inclusion criteria were excluded. Included 177 participants were further assessed for tissue availability for immunohistochemistry staining. Finally, 68 participants with both tumor and non-neoplastic renal tissue available were included in the paired-t test. Participants with the tumor tissue available (n = 114) or with the perinephric adipose tissue available (n = 60) were included in the binary logistic regression analysis.

This study received ethics approval from the Metro South Human Research Ethics Committee (HREC/05/QPAH/95; HREC/16/QPAH/353) and utilised archived formalin-fixed, paraffin-embedded (FFPE) specimens from consenting patients who underwent nephrectomy for renal tumors at the Princess Alexandra Hospital between June 2013 and September 2017 [12]. Participants had provided consent for the anonymous use of their clinical data and tissue samples, including renal tumor tissue, non-neoplastic renal tissue and perinephric adipose tissue. Inclusion criteria include that, 1) aged over 18 years old; 2) did not accept treatment for kidney cancer before surgery and 3) diagnosis of ccRCC. Following nephrectomy, samples of tumor tissue, distal non-neoplastic cortical tissue, and tumor-adjacent perinephric adipose tissue were excised, fixed in 4% formaldehyde for 24 hours, and stored at 4°C prior to being blocked in paraffin. Clinical data were included in a database. Demographic data, medical and smoking history were collected from structured interviews and corroborated through chart review. Pathological diagnosis was recorded from the pathology report. Tumors were staged according to the 7^th^ TNM Classification of Malignant Tumors [13] and graded according to the International Society for Urological Pathology (ISUP) grading system for RCC by two pathologists [14]. Work flow of study participation is summarized in Fig 1.

### IHC staining

**Fig 2:**
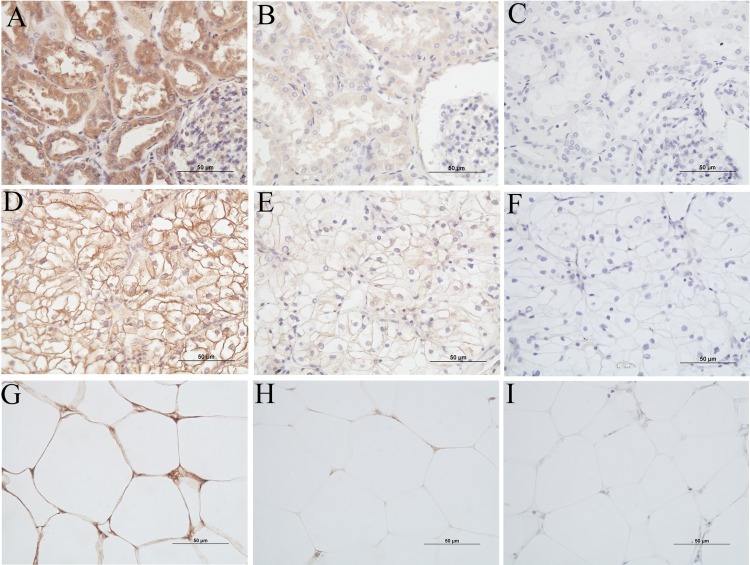
Staining patterns of GRP78 in different tissues. Images were captured at x40 using Nikon Brightfield microscopy. (A) Non-neoplastic renal tissue; (B) Negative control of non-neoplastic renal tissue (blocking peptide mixed with primary antibody); (C) Negative control of non-neoplastic renal tissue (eliminating primary antibody); (D) Clear cell renal cell carcinoma; (E) Negative control of clear cell renal cell carcinoma (blocking peptide mixed with primary antibody); (F) Negative control of clear cell renal cell carcinoma (eliminating primary antibody); (G) Perinephric adipose tissue; (H) Negative control of perinephric adipose tissue (blocking peptide mixed with primary antibody); (I) Negative control of perinephric adipose tissue (eliminating primary antibody).

Sections of 4μm thickness were cut onto Superfrost slides. Paraffin was removed by xylene and the tissue was rehydrated through graded washes of ethanol in water, ending in a final rinse in water. Endogenous peroxidase activity was quenched by incubation in 3% H_2_O_2_ (H-1009, Sigma) for 10 min. A microwave (Whirlpool, 850W) was used for antigen retrieval. Slides were put into a lidded glass container which was filled with 250mL of Tris-EDTA (10Mm Tris Base, 1Mm EDTA solution, 0.05% Tween 20, pH9) solution. Two cycles of antigen retrieval (first cycle: power of 10 for 2 min and 20s; second cycle: power of 2 for 15 min) were applied. Non-specific binding was blocked by Background Sniper (BS966, Biocare Medical) for 15 min, followed by an incubation with the primary antibody (GRP78, 1:50, SANTSC-376768, Santa Cruz Biotechnology) at room temperature for one hour. MACH 1 Universal HRP-Polymer detection kit (901-M1U539-082914, Biocare Medical) containing anti-mouse secondary antibody (incubated for 15 min), signal amplification reagent (incubated for 30 min) and diaminobenzidine hydrochloride chromogen (DAB) (incubated for 5 min), was used per manufacturer’s instructions followed by counterstaining with haematoxylin (AHH-1L ProSciTech). A section of liver tissue (gifted by Clay Winterford, Berghofer Medical Research Institute, Brisbane) known to express GRP78, as indicated by the primary antibody datasheet, was used as the positive control. The negative control was done by eliminating the primary antibody prior to adding the secondary antibody. The specificity of the GRP78 antibody was tested by mixing the primary antibody with a blocking peptide (GL Biochem/Shanghai-687830, amino acid sequence: ee edkkedvgtv vgidlgttys cvgvfkngrv) at a concentration ten times of the primary antibody. The mixture was left at room temperature for one hour before being added to the slides (Fig 2). Staining of slides was carried out blinded to the medical records.

### Image capture and analysis

The stained slides were scanned with Olympus Slide Scanner VS120 (rm4026,) using the x20 objective. The scanned images were viewed using the OlyVia image reading software. Three to five snapshots were captured randomly at 50% view size from each original image. The cropped images were saved in TIFF format and exported into the software Fiji for analysis [15]. Firstly, a statistical model was built using IHC toolbox to detect the DAB-stained color in renal tissues and adipose tissues [16]. Secondly, images were manually edited by deleting glomeruli, large blank areas, vessels and fibrosis. Macro language was written for processing series of commands. Color threshold was set to “0–254” prior to measuring average grey value and proportion of positive pixels. Methods used for measuring the proportion of positive pixels were based on previous studies [17]. Average grey value was used to measure staining intensity of DAB. The more chromogen present in stained areas, the darker the brown color appeared macroscopically. However, as measured by the standard intensity function of Fiji, standard red-green-blue (RGB) color images acquired from bright field microscopy had the lowest average grey value of 0 for a black, dark-stained area and the highest average grey value of 250 for a white, unstained area [18]. This resulted in an inverse correlation between average grey value and staining intensity. Assessment of slides was carried out blinded to the medical records.

### Statistical analysis

Categorical data were reported as count (percentage). Continuous data were reported as mean (95% confidence interval [CI] or standard deviation) or median (interquartile range [IQR]), depending on whether or not the data were normally distributed. Outcome variables regarding ccRCC aggressiveness were dichotomized as follows: tumor grade: low [1–2] and high [3–4]; tumor stage: low [1–2] and high [3–4]; tumor size: [≤70mm] and [>70mm]; metastases: [presence] and [absence]. A paired-t test was used to compare GRP78 expression (measured by both average grey value of staining and proportion of positively stained pixels) between renal tumor tissue and adjacent non-neoplastic renal tissue. Binary logistic regression was applied to model ccRCC aggressiveness based on the expression value of GRP78, adjusting for potential covariates, including BMI, history of smoking and diagnosis of hypertension. To evaluate the predictive ability of the logistic models, receiver operating characteristic (ROC) curves were fitted to the logistic models and the area under the ROC curve (AUC) reported. Differences in GRP78 expression between benign tumor and ccRCC, and distribution of GRP78 between different outcome variables were displayed descriptively. P-values<0.05 were considered statistically significant. Statistical analysis was performed using Stata 14 (StataCorp, College Station, TX, USA).

## Results

### Patient characteristics

267 participants recorded in the database were assessed for eligibility. After excluding 90 participants who did not meet the inclusion criteria (diagnosis other than clear cell renal cell carcinoma n = 85, missing data about kidney cancer diagnosis n = 5), 177 participants were further assessed for tissue availability when included in specific statistical analysis. 68 participants with both tumor and non-neoplastic renal tissue available for IHC were included in the paired-t test when comparing the GRP78 expression between tumor tissue and the matched non-neoplastic renal tissue. Participants with the tumor tissue available (n = 114) or with the perinephric adipose tissue available (n = 60) for IHC were included in the binary logistic regression analysis when predicting ccRCC aggressiveness based on the expression value of GRP78 in either tissue type (Fig 1). Demographic and clinicopathological characteristics of 68 participants for whom both tumor tissue and normal renal tissue were available for IHC analysis are displayed in Table 1.

**Table 1:** Characteristics of the study population with both ccRCC tumor and non-neoplastic renal tissue available for immunohistochemistry.

Variables	Study cohort (n = 68)
Age (years), mean (sd)	61 (11)
Age <60 years	32 (47%)
Female	27 (40%)
White race^a^	65 (98%)
Diabetes^a^	13 (19%)
Hypertension^a^	43 (65%)
Smoking history^a^	
Former and current	37 (57%)
Never	28 (43%)
BMI (kg/m^2^)^a^, mean (sd)	30 (7)
BMI category^a^	
Underweight (<18.5 kg/m^2^)	2 (3%)
Normal weight (18.5–24.9 kg/m^2^)	11 (17%)
Overweight (25–29.9 kg/m^2^)	27 (42%)
Obese (≥30 kg/m^2^)	25 (38%)
eGFR (ml/min/1.72m^2^), mean (sd)	77 (18)
CKD stage	
1 (eGFR >90 mL/min/1.73 m^2^)	14 (21%)
2 (eGFR 60–90 mL/min/1.73 m^2^)	43 (63%)
3 (eGFR 30–59.9 mL/min/1.73 m^2^)	11 (16%)
4 or 5 (eGFR <30 mL/min/1.73 m^2^)	0
Tumor size (mm), median [IQR]	50 [36, 74]
Tumor size >70 mm	18 (26%)
Tumor stage^a^	
1	32 (48%)
2	4 (6%)
3	29 (43%)
4	2 (3%)
Tumor grade	
1	3 (4%)
2	24 (35%)
3	29 (43%)
4	12 (18%)
Presence of distant metastasis^a^	9 (13%)
Proportion of positive pixels in tumor tissues, mean (sd)	0.47 (0.12)
Proportion of positive pixels in normal tubules, mean (sd)	0.44 (0.10)
Staining intensity in tumor tissues, mean (sd)	202.0 (3.6)
Staining intensity in normal tubules, mean (sd)	202.8 (4.0)

^a^Missing data ≤4%

Abbreviations: BMI, body mass index; CKD, chronic kidney disease; ccRCC, clear cell renal cell carcinoma; eGFR, estimated glomerular filtration rate; sd, standard deviation

### Staining patterns of GRP78 in tumor tissue and non-neoplastic renal tissue

**Fig 3:**
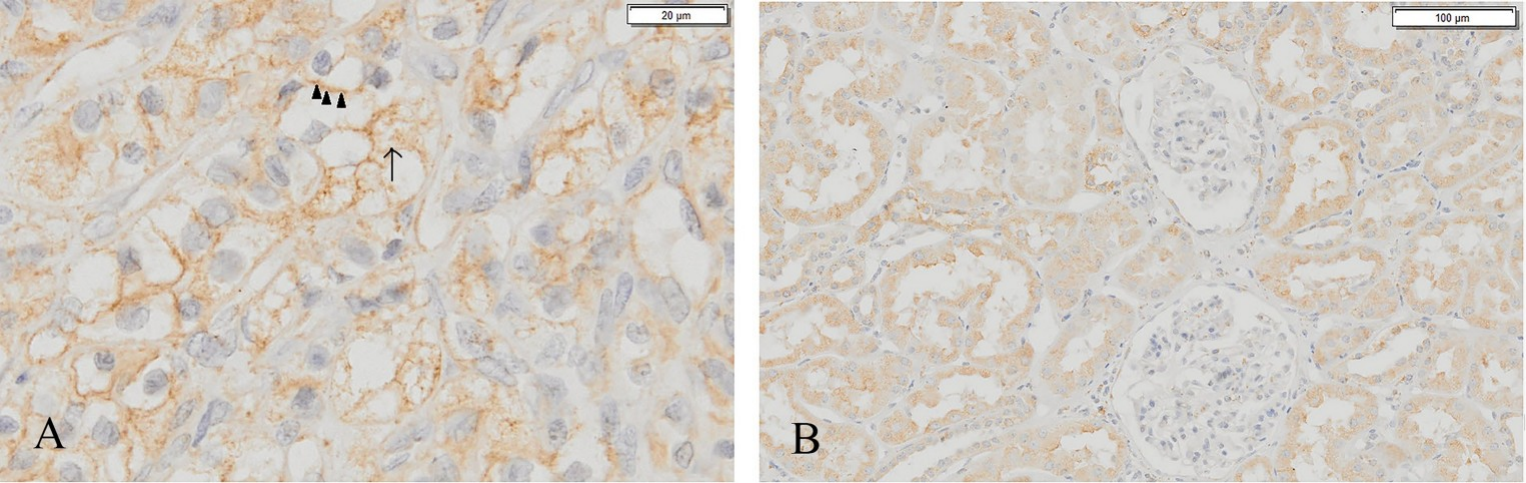
Staining patterns of GRP78 in ccRCC tumor tissue and non-neoplastic renal tissue. (A) Image was captured at x40 under 100% view. Arrow: staining on the cytoplasma; arrow heads: staining close to the cell membrane; (B) Image was captured at x10 under 25% view.

The expression of GRP78 is mainly observed in areas close to the cell membrane. Some cytoplasma of ccRCC cells were left unstained due to the lipid-rich cytoplasmic deposits being washed away during tissue processing **(**Fig 3A**)**. In non-neoplastic renal tissue, GRP78 is positive in all tubules with no distinctive difference of staining intensity between proximal renal tubule and distal renal tubule epithelial cells. However, in the capillary bundles of glomeruli, the GRP78 expression is much lower (Fig 3B).

### Difference of GRP78 expression in renal tumor and normal renal tissues

Under both quantification methods, the difference in GRP78 expression between tumor renal and paired non-neoplastic renal tissue was modest (staining intensity method: mean difference = -0.74, (95% CI: -1.67 to 0.19, p = 0.12; positive pixel method: mean difference = 0.03, 95% CI: - 0.003 to 0.06, p = 0.07; Table 2).

**Table 2:** Difference of GRP78 expression between ccRCC tumor and adjacent non-neoplastic renal tissues (n = 68).

	Tumor tissues	Adjacent non-neoplastic renal tissues	95% confidence interval for mean difference	p-value
	mean (sd)	mean (sd)		
Average grey value	202.04 (3.64)	202.78 (3.85)	-1.67 to 0.19	0.12
Proportion of positive pixels	0.47	0.44	-0.003 to 0.06	0.07

There was a statistically insignificant smaller average grey value (interpreted as stronger staining intensity) in renal tumor tissues when it was compared with the paired non-neoplastic renal tissues (mean difference = -0.74, p = 0.12). There was a higher proportion of positive pixels in renal tumor tissues when it was compared with a paired non-neoplastic renal tissue, which did not reach statistical significance (mean difference = 0.03, p = 0.07). Abbreviation: sd, standard deviation

### Association of GRP78 expression in tumor tissue with ccRCC aggressiveness

**Fig 4:**
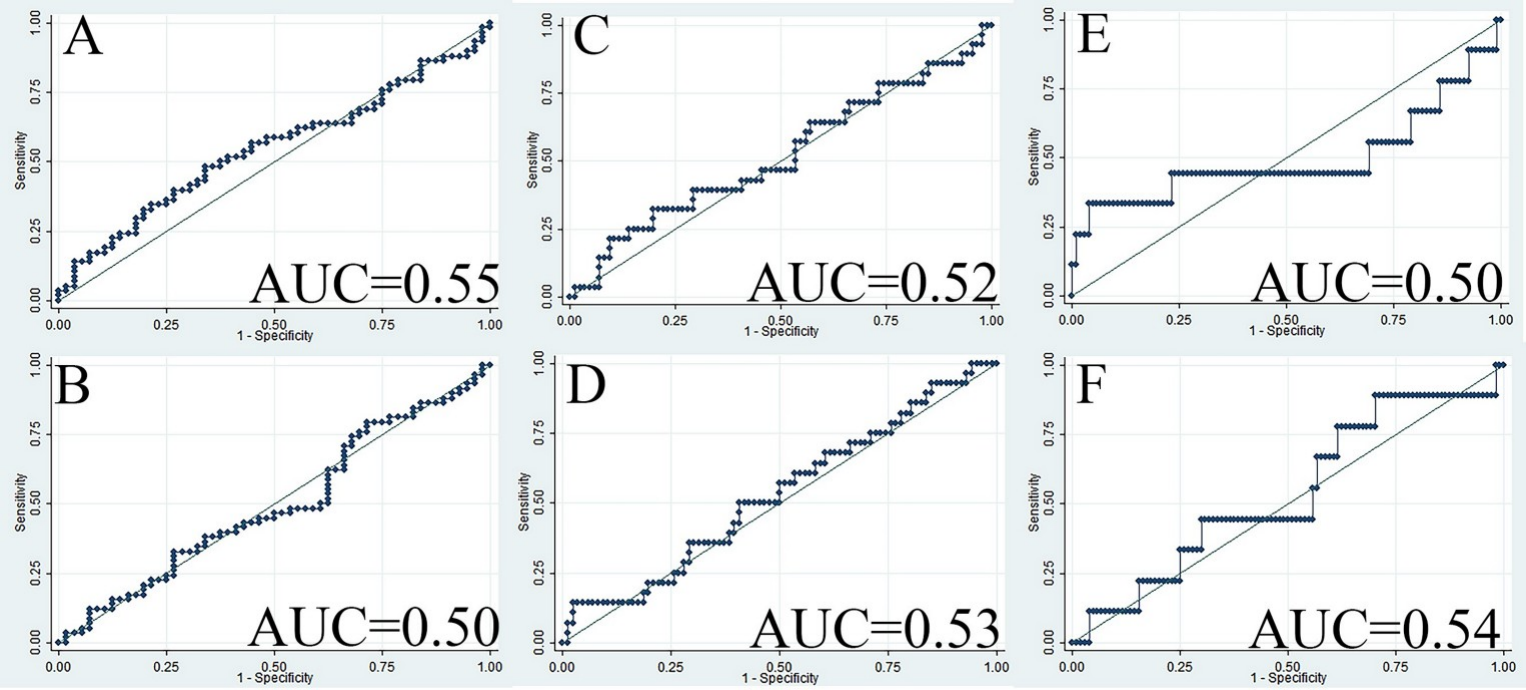
ROC curves when GRP78 expression in ccRCC tumor tissue was applied to predict ccRCC aggressiveness. (A) ROC curve when staining intensity in tumor tissue was applied to predict tumor grade (OR = 0.96, AUC = 0.55); (B) ROC curve when proportion of positive GRP78 staining in tumor tissue was applied to predict tumor grade (OR = 0.95, AUC = 0.50); (C) ROC curve when staining intensity in tumor tissue was applied to predict tumor size (OR = 0.99, AUC = 0.52); (D) ROC curve when proportion of positive GRP78 staining in tumor tissue was applied to predict tumor size (OR = 4.26, AUC = 0.53); (E) ROC curve when staining intensity in tumor tissue was applied to predict whether there is a presence of distant metastasis (OR = 1.08, AUC = 0.50); (F) ROC curve when proportion of positive GRP78 staining in tumor tissue was applied to predict whether there is a presence of distant metastasis (OR = 1.93, AUC = 0.54). Abbreviations: AUC, area under the curve; ccRCC, clear cell renal cell carcinoma; OR, odds ratio; ROC, receiver operating characteristics.

Distribution of GRP78 expression in tumor tissue among different categories of the outcome variables is summarized in Table 3. When adjusting for BMI, hypertension and history of smoking, we did not find any tumor grading categorizing potential for either staining intensity measurement (adjusted odds ratio [aOR] = 0.99, 95% CI: 0.88 to 1.12, AUC = 0.55, Fig 4A) or proportion of positive pixel measurement (aOR = 0.56, 95% CI: 0.02 to 17, AUC = 0.50, Fig 4B). Since high tumor stage was largely ascribed by large tumor size (aOR = 16.6, 95% CI: 5.3 to 52.0) and presence of metastasis (aOR = 2.5, 95% CI: 0.36 to 17.5), tumor size and metastasis were set as two separate outcome variables to describe ccRCC aggressiveness. Neither staining intensity (aOR = 0.98, 95% CI: 0.84 to 1.14) nor proportion of positive pixels (aOR = 3.8, 95% CI: 0.07 to 217.9) was found to have any predictive potential for tumor size with ROC curves showing poor discrimination ability (AUC < 0.54, Fig 4C and 4D). Similar results were observed for metastasis for both staining intensity (aOR = 1.12, 95% CI: 0.88 to 1.41, Fig 4E) and proportion of positive pixels (aOR = 1.39, 95% CI: 0.00 to 814.4, Fig 4F).

**Table 3:** Distribution of GRP78 expression in ccRCC tumor tissue (N = 114) among different categories of ccRCC aggressiveness.

ccRCC aggressiveness categories	N	Staining intensity	95% CI	Proportion of positive pixels	95% CI
Tumor grade					
Grade1	13	202.6	[201.2,204.0]	0.49	[0.42,0.56]
Grade2	43	202.5	[201.6,203.3]	0.47	[0.44,0.51]
Grade3	42	201.8	[200.7,202.9]	0.49	[0.45,0.53]
Grade4	16	202.9	[200.7,205.0]	0.45	[0.39,0.51]
Tumor stage^a^					
Stage 1	64	202.4	[201.7,203.1]	0.47	[0.44,0.50]
Stage 2	7	201.8	[199.7,203.9]	0.47	[0.40,0.53]
Stage 3	39	202.6	[201.5,203.8]	0.48	[0.44,0.52]
Stage 4	3	196.3	[183.7,208.8]	0.53	[0.26,0.84]
Tumor size					
≤70mm	86	202.3	[201.7,203.0]	0.47	[0.45,0.50]
>70mm	28	202.2	[200.8,203.5]	0.49	[0.45,0.54]
Distant metastasis^a^					
No distant metastasis	104	202.22	[201.7,202.8]	0.48	[0.45,0.50]
Presence of distant metastasis	9	203.1	[198.3,207.8]	0.49	[0.38,0.59]

^a^Missing data <1%

### Association of GRP78 expression in perinephric adipose tissue with ccRCC aggressiveness

**Fig 5:**
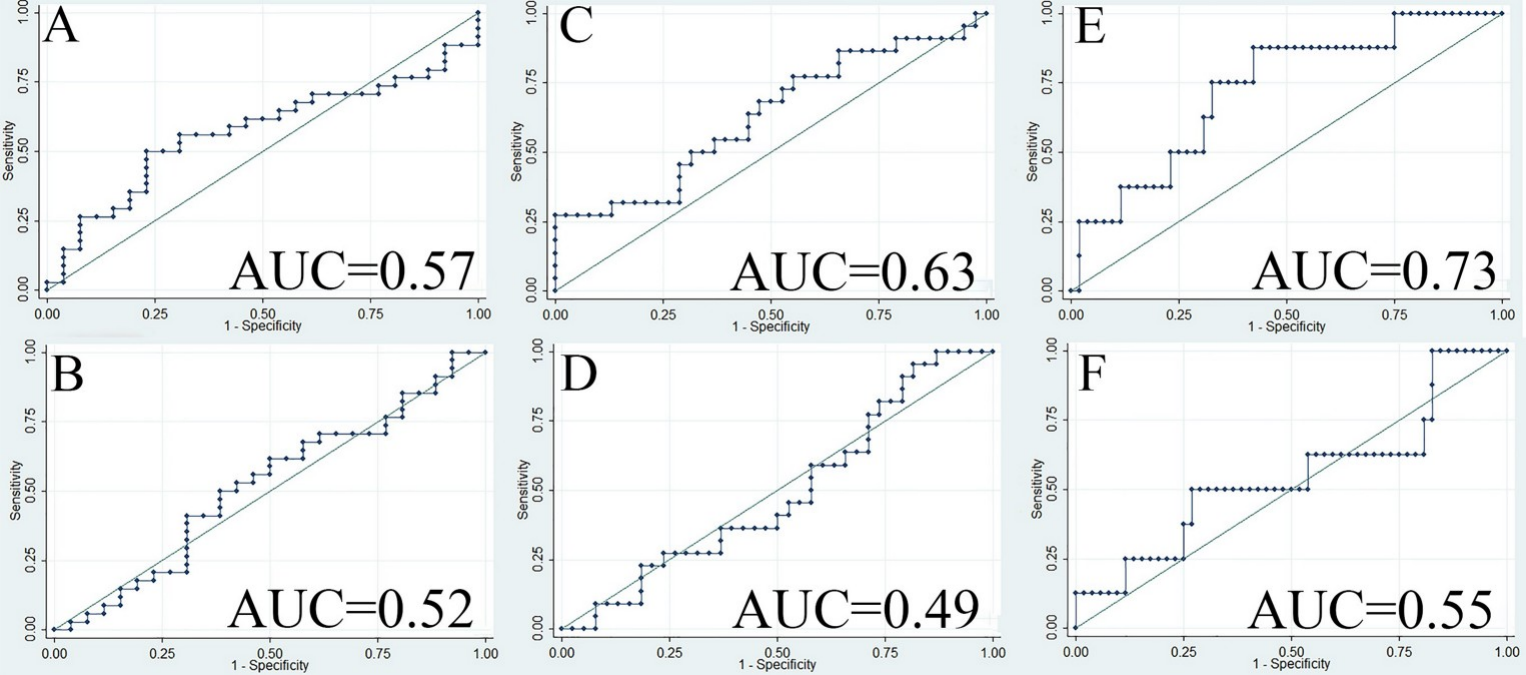
ROC curve when GRP78 expression in perinephric adipose tissue was applied to predict the presence of distant metastasis. (A) ROC curve when staining intensity in adipose tissue was applied to predict tumor grade (OR = 1.01, AUC = 0.57); (B) ROC curve when proportion of positive GRP78 staining in adipose tissue was applied to predict tumor grade (OR = 1.00, AUC = 0.52); (C) ROC curve when staining intensity in adipose tissue was applied to predict tumor size (OR = 1.08, AUC = 0.63); (D) ROC curve when proportion of positive GRP78 staining in adipose tissue was applied to predict tumor size (OR = 0.99, AUC = 0.49); (E) ROC curve when staining intensity in adipose tissue was applied to predict whether there is a presence of distant metastasis (OR = 1.17, AUC = 0.73); (F) ROC curve when proportion of positive GRP78 staining in adipose tissue was applied to predict whether there is a presence of distant metastasis (OR = 1.06, AUC = 0.55). Abbreviations: AUC, area under the curve; ccRCC, clear cell renal cell carcinoma; OR, odds ratio; ROC, receiver operating characteristics.

Distribution of GRP78 expression in adipose tissue among different categories of the outcome variables is summarized in Table 4. Similar to the results in the tumor tissue, neither staining intensity (aOR = 1.00, 95% CI: 0.91 to 1.11, AUC = 0.57, Fig 5A) nor proportion of positive pixels (aOR = 0.95, 95% CI: 0.84 to 1.08, AUC = 0.52, Fig 5B) in the adipose tissue was found to have any tumor grading categorizing potential. Similarly, neither staining intensity (aOR = 1.00, 95% CI: 0.79 to 1.25, AUC = 0.63, Fig 5C) nor proportion of positive pixels (aOR = 0.95, 95% CI: 0.84 to 1.07, AUC = 0.49, Fig 5D) was found to have any predictive potential for tumor size. Regarding the metastatic discrimination potential, for one unit increase in average grey value of GRP78 staining intensity in perinephric adipose tissue, the odds of ccRCC being diagnosed with distant metastasis increased by 17% (95% CI: 0.99 to 1.38, AUC = 0.73, Fig 5E). However, no metastatic discrimination potential was found for proportion of positive pixels (OR = 1.06, 95% CI: 0.91 to 1.23, AUC = 0.55, Fig 5F).

**Table 4:** Distribution of GRP78 expression in ccRCC associated perinephric adipose tissue (N = 60) among different categories of ccRCC aggressiveness.

ccRCC aggressiveness categories	N	Staining intensity	95% CI	Proportion of positive pixels	95% CI
Tumor grade					
Grade 1	1	190.7	-	0.07	-
Grade 2	25	185.2	[183.4,187.0]	0.13	[0.10,0.15]
Grade 3	21	183.8	[180.1,187.5]	0.13	[0.11,0.15]
Grade 4	13	189.3	[187.5,191.1]	0.12	[0.09,0.14]
Tumor stage					
Stage 1	28	184.2	[181.9,186.6]	0.13	[0.11,0.15]
Stage 2	3	187.2	[173.7,200.7]	0.16	[0.05,0.27]
Stage 3	27	187.1	[184.6,189.6]	0.11	[0.10,0.13]
Stage 4	2	185.1	[164.3, 205.8]	0.14	[-0.09,0.37]
Tumor size					
≤70 mm	38	184.8	[182.9,186.7]	0.13	[0.11,0.14]
>70 mm	22	187.3	[184.4,190.1]	0.12	[0.11,0.14]
Distant metastasis					
No distant metastasis	52	185.1	[183.4,186.8]	0.12	[0.11,0.14]
Presence of distant metastasis	8	189.3	[186.0,192.7]	0.14	[0.09,0.19]

### Difference of GRP78 staining intensity in ccRCC associated adipose tissues and benign tumor associated adipose tissues

**Fig 6:**
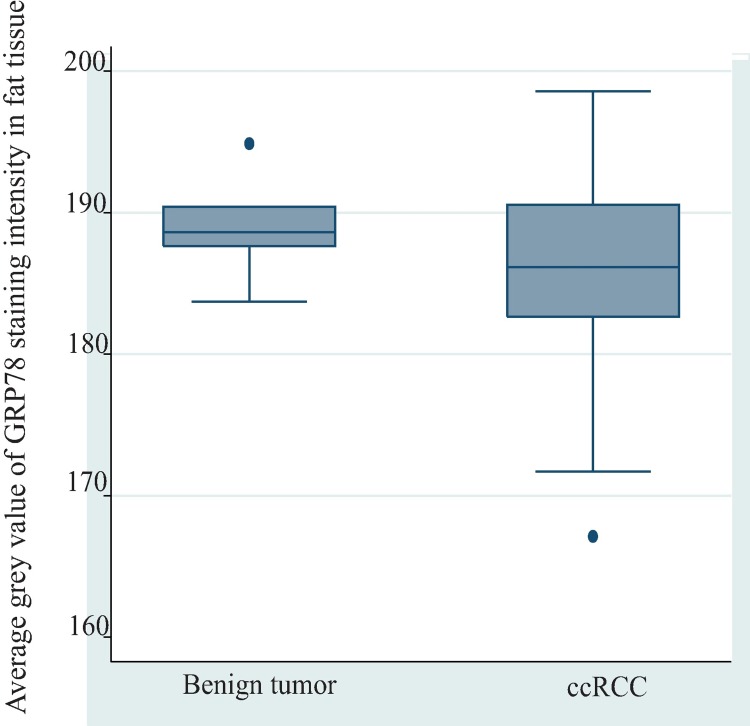
Difference of GRP78 staining intensity between benign tumor associated adipose tissue and ccRCC associated adipose tissue. There was a higher expression of GRP78 in ccRCC-associated adipose tissue (N = 60) compared with that in benign tumor-associated adipose tissue (N = 7), as indicated by a lower average grey value in ccRCC tumor associated adipose tissue than benigh tumor-assoicated adipose tissue (mean difference of average grey value = -3.26, P = 0.17). Abbreviation: ccRCC, clear cell renal cell carcinoma.

**Fig 7:**
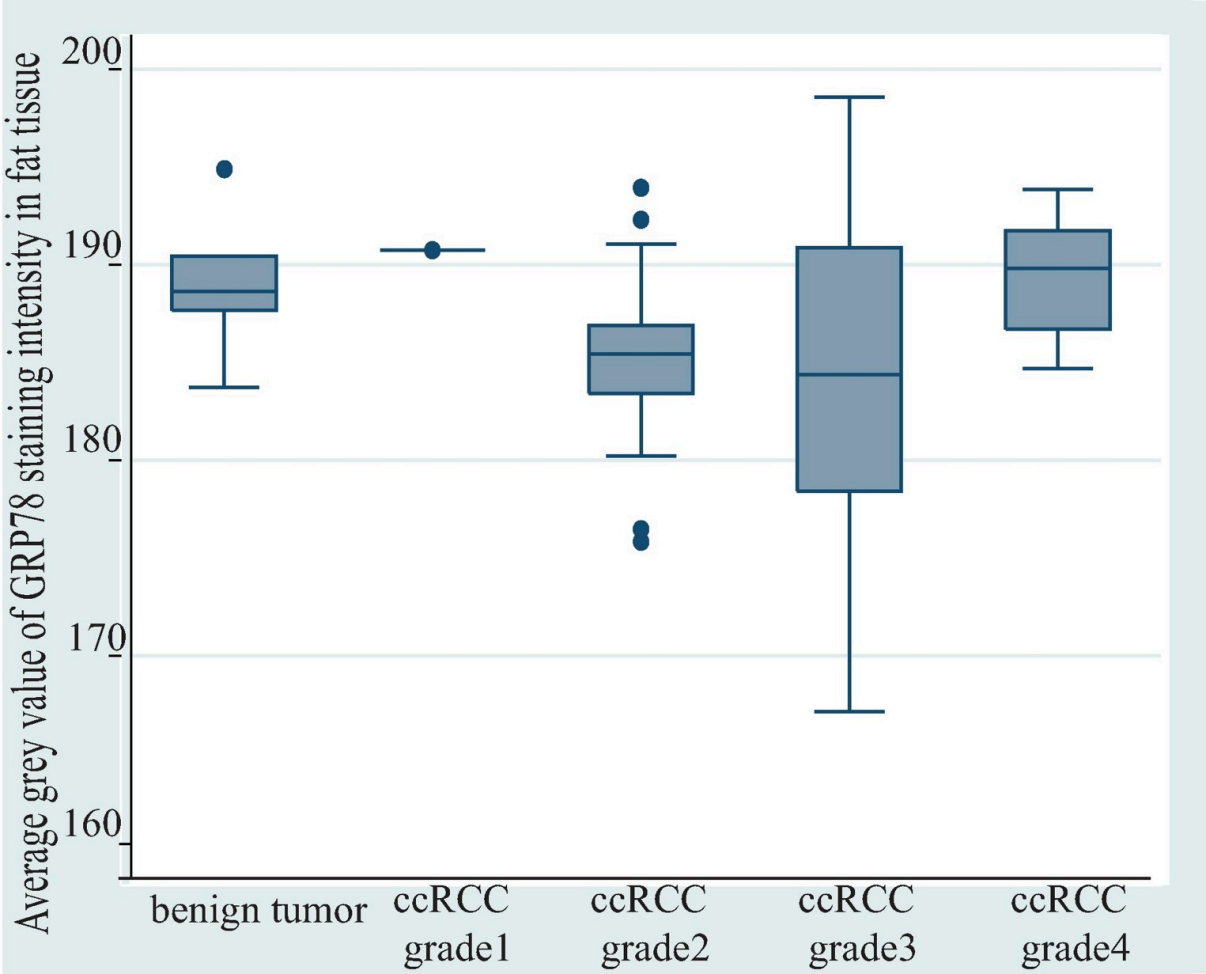
Distribution of GRP78 staining intensity in tumor associated adipose tissue among benign tumor and different ccRCC grades. Compared with grade 2 (N = 25) and grade 3 (N = 21) ccRCC, grade 4 ccRCC (N = 13) and benign tumor (N = 7) exhibited a lower expression of GRP78, as indicated by greater average grey values. Abbreviation: ccRCC, clear cell renal cell carcinoma.

The GRP78 staining intensity between ccRCC and benign tumor associated adipose tissues were compared to examine the risk stratifying potential of GRP78 expression in perinephric adipose tissue (Fig 6). There was no statistically significant difference in expression of GRP78 between ccRCC-associated (N = 60) and benign tumor-associated adipose tissue (N = 7) (mean difference of average grey value = -3.26, p = 0.17). The distribution of GRP78 staining intensities in tumor-associated adipose tissues among benign tumors and different grades of ccRCC were further explored (Table 5). The box plot (Fig 7) demonstrated that, compared with grade 2 and grade 3 ccRCC, grade 4 ccRCC and benign tumors exhibited a lower expression of GRP78.

**Table 5:** Distribution of GRP78 staining intensity in benign tumor-associated adipose tissue and ccRCC associated perinephric adipose tissue.

	N	Staining intensity	95% CI
ccRCC tumor grade			
Grade 1	1	190.7	-
Grade 2	25	185.2	[183.4,187.0]
Grade 3	21	183.8	[180.1,187.5]
Grade 4	13	189.3	[187.5,191.1]
Benign tumor^a^	7	188.9	[185.8,192.0]

^a^ Five oncocytoma and two non-neoplastic cystic benign tumor were combined into benign tumor.

Abbreviation: ccRCC, clear cell renal cell carcinoma

## Discussion

In this study, GRP78 expression was not upregulated in ccRCC tumor tissue compared with the paired normal renal tissue. Moreover, GRP78 expression levels in both renal tumor tissue and tumor-associated perinephric adipose tissue were not associated with grade or size of ccRCC tumors, except for a weak metastatic predictive potential that was found for GRP78 staining intensity in ccRCC tumor-associated perinephric adipose tissue. However, due to the number (N = 8) in the least frequent outcome variable (presence with metastasis) being less than 10 (Table 4), no covariate was introduced in the model to adjust bias. Moreover, this finding was not supported when proportion of positive pixels was applied to measure GRP78 expression. Although descriptive analysis found benign tumor-associated adipose tissue exhibited higher average grey value of GRP78 staining intensity than ccRCC-associated adipose tissue (Fig 6), it is not reasonable to deduce weak GRP78 staining intensity (as demonstrated by higher average grey value) is related to a better prognosis, due to an even weaker GRP78 staining intensity that was found in grade 4 ccRCC tumor-associated adipose tissue (Fig 7).

**Fig 8:**
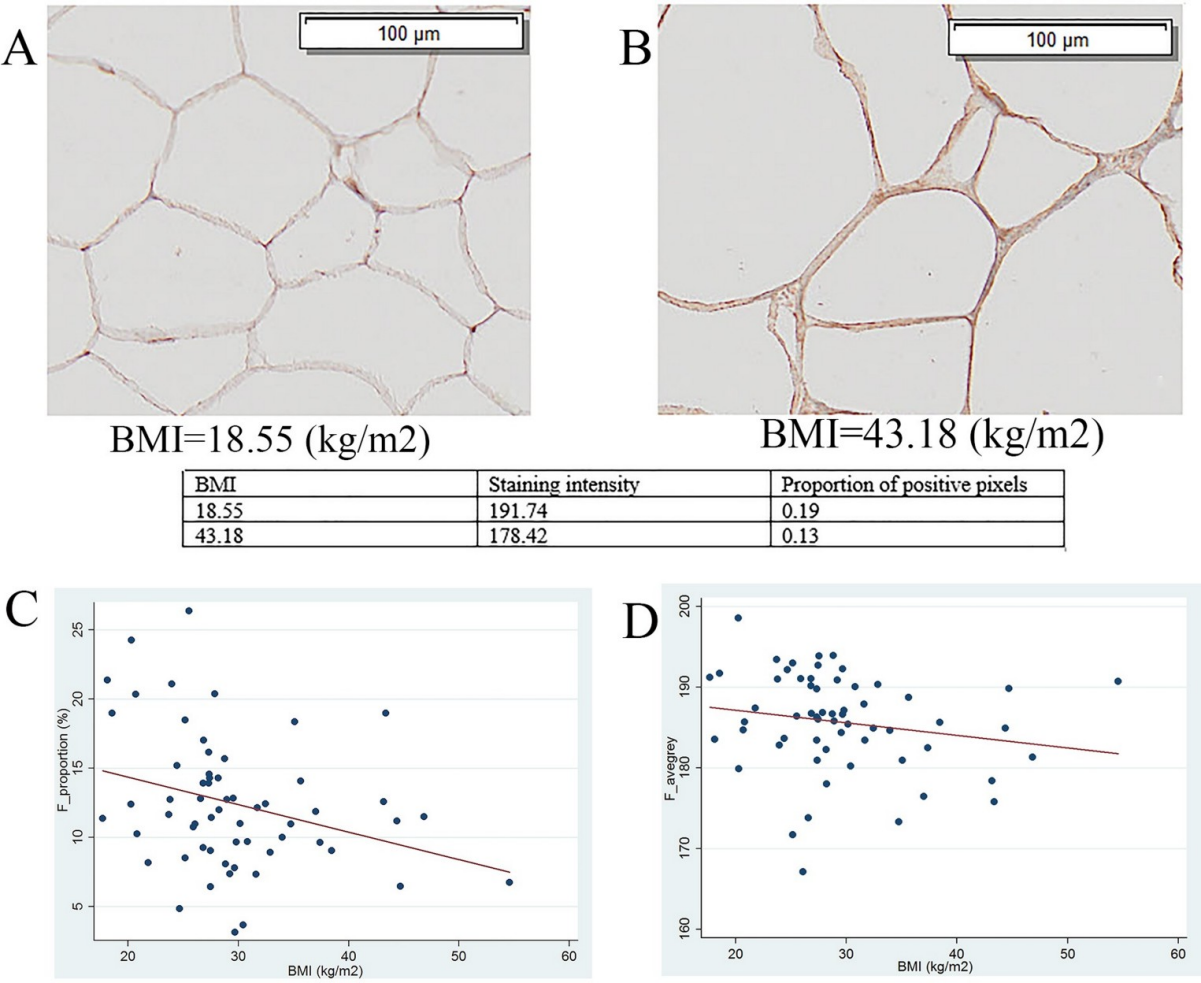
The impact of adipose tissue heterogeneity on proportion of positive pixels. (A) Images were captured at ×10 under 50% view. BMI = 18.55; Average grey value = 191.74; Proportion of positive pixels = 0.19; (B) Images were captured at ×10 under 50% view. BMI = 43.18; Average grey value = 178.42; Proportion of positive pixels = 0.13; (C) Scatter plot of BMI versus proportion of positive GRP78 stained pixels in adipose tissue. Coefficient = -0.20, P = 0.02; (D) Scatter plot of BMI versus average grey value of GRP78 staining in adipose tissue. Coefficient = -0.16, P = 0.15. Abbreviation: BMI, body mass index; F_avegrey, average grey value of GRP78 staining in adipose tissue; F_proportion, proportion of positive GRP78 stained pixels in adipose tissue.

Contrary to the findings by Fu *et al*. [5], we could not find upregulation of GRP78 expression in renal tumor tissue. This apparent disparity may be explained by methodologic differences in the approach to accounting for tissue heterogeneity. Compared with ccRCC tumor tissue, the normal nephron has much greater tissue heterogeneity, being composed of glomeruli and tubules with lumens of various size [19]. Obviously, the tubular lumen will remain unstained in IHC. Theoretically, glomeruli, the major components of which are capillary bundles, will be more weakly stained by GRP78 than the rest of the nephron structure, because the ER does not exist in erythrocytes [20]. Hence, failure to appropriately account for tissue heterogeneity may introduce bias when comparing the proportion of positively stained areas between renal tumor tissue and paired normal renal tissue. However, the previous publication did not use a method that mitigated the risk of the bias of tissue heterogeneity. On the other hand, in the present study, tubular lumens and glomeruli were manually eliminated prior to comparing the proportions of GRP78 positively stained areas between tumor and adjacent non-neoplastic renal tissue. Similarly, the conflicting results between staining intensity and proportion of positive pixels may be partially explained by adipose tissue heterogeneity as a result of variation in size of adipocytes. For example, larger adipocytes for participants with higher BMI may leave the cellular stainable area smaller (lipid being washed from cells during processing) (Fig 8A and 8B), which was consistent with the statistically significant inverse association observed between BMI and proportion of positive pixels (r = - 0.18, p = 0.02) (Fig 8C). However, we did not find any significant association between BMI and staining intensity (r = -0.14, p = 0.13) (Fig 8D).

The hypoxic cancer microenvironment suppresses the differentiation of tumor cells. Likewise, undifferentiated tumor cells tend to grow faster, exacerbating hypoxia within the cancer microenvironent [21]. The activation of ER stress is an adaptive behavior in response to such a suboptimal microenvironment [22]. The observed adipose tissue GRP78 staining intensity generally met with this supposition, showing a gradually increasing staining intensity from grade 0 to grade 3. Opposite to an expected over expression of GRP78, the GRP78 staining intensity was the lowest in grade 4 ccRCC (Fig 7). It is difficult to interpret the result further, due to small sample size and the lack of examination of the whole ER stress signaling network.

This study has the largest sample size to date and was based on the investigation of a different race population compared with the previously published study by Fu and colleagues. Other strengths of this study include use of appropriate statistical tests and software-assisted DAB chromogen quantification of both average grey value of GRP78 staining intensity and proportion of positive pixels. On the other hand, the findings in this study are subjected to two major limitations. Firstly, this study only examined the expression of one ER stress marker, GRP78. Although the activation of GRP78 is considered as an initial sign of ER stress, the one time-point assessment of GRP78 expression cannot reflect the full-course status of ER stress [23], because ER stress involves mutiple downstream signaling pathways that are mediated by different signalling proteins [24]. Secondly, quantifying DAB chromogen intensity in IHC has long been a controversial issue, because the brightness of a DAB-stained region is not directly proportional to the concentration of localized antigen [23]. However, this is an inherent limitation of the IHC stainining. In the process of image analysis, we found the result of average grey value fit with the intuitive impression of the staining intensity.

Collectively, the findings of the present study failed to demonstrate any utility of GRP78 as a risk stratifying marker for ccRCC. This does not necessarily refute a role for activated ER stress as a potential therapeutic target for kidney cancer, because the peak expression of different ER stress markers may vary with different stages in cancer development [25]. Further studies in this area are warranted.

## Conclusion

GRP78 is not upregulated in renal tumor tissue compared with paired normal renal tissue. Thus, for the purpose of risk stratification of ccRCC, GRP78 would not appear to be a useful marker. The predictive value of GRP78 expression in adipose tissue is uncertain due to the presence of conflicting findings according to methodologic approach.

## Supporting information

S1 Table Dataset. The raw data is available in S1 Table. The raw dataset recorded demographic information of participants, such as age, sex (M for male and F for female), ethinicity, body mass index, smoking history and diagnosis. For smoking history, the code of “1” represents “current smoker”; the code of “0” represents “never smoked”; the code of “10” represents “smoking quitted”. For diagnosis of diabetes and hypertension, the code of “1” represents “diagnosed with diabetes or hypertension”; the code of “0” represents “not diagnosed with diabetes or hypertension”. The dataset also included pathological diagnosis of renal tumors, such as tumor subtypes, tumor stage, tumor grade, tumor size and whether there was a presence of metastasis (the code of “1” represents “diagnosed with distant metastasis”; the code of “0” represents “not diagnosed with distant metastasis”). Additionally, the average grey value and proportion of positive pixels of the staining in different tissues were presented. Abbreviation: T_proportion: proportion of positive pixels in tumor tissues; T_avegrey: staining intensity in tumor tissues; N_proportion: proportion of positive pixels in normal renal tissues; N_avegrey: staining intensity in normal renal tissues; F_proportion: proportion of positive pixels in fat tissues; F_avegrey: staining intensity in fat tissues.
(XLSX)
